# Clinical outcomes after intentional replantation of permanent teeth: A systematic review

**DOI:** 10.17305/bjbms.2019.3937

**Published:** 2020-02

**Authors:** Lin Wang, Hua Jiang, Yang Bai, Qiang Luo, Hao Wu, Hongchen Liu

**Affiliations:** Department of Stomatology, Chinese PLA General Hospital, Beijing, China

**Keywords:** Intentional replantation, tooth, survival rate

## Abstract

This study aimed to systematically assess the outcomes of intentional replantation (IR) of teeth and to determine the survival rate, success rate, and prognostic factors related to the treatment. A search was conducted for all relevant English language articles published from January 2000 to October 2017. The search terms included “intentional replantation” and “teeth” according to the inclusion criteria. The Methodological Index for Non-randomized Studies (MINORS) was used to assess the methodological quality of included studies. Twelve studies were identified as relevant for the systematic review. In total, 896 patients with 905 teeth were examined for intentional teeth replantation. The success rate was greater than 90% in four studies (33.33%) and between 70% and 80% in five studies. At short-term follow-up (<6 months), the survival rate was approximately 90% in four studies. At longer-term follow-up (>36 months), the survival rates of teeth were slightly reduced in four studies, and tended to be stable after 48 months. In conclusion, the long-term success and survival rate of IR are likely dependent upon short extraoral time, reduced pocket depth, type of tooth, type of root-end filling material, and the prevention of atraumatic tooth root damage.

## INTRODUCTION

Intentional replantation (IR) is a concept that has been known for over a thousand years. It is defined as the intentional removal of a tooth and reinsertion into the extraction socket before or after proper endodontic treatment, and is regarded as the last option for the treatment of periodontitis, pulpitis and post-trauma [1-5]. Previously, complete procedure guidelines for IR were not available, and a high probability of success was not expected. The criteria for IR have evolved gradually over time. In 1966, Grossman’s team reported a series of indications, including iatrogenic or natural indications, canal obstruction and complex anatomy [6]. In a systematic review, only 8 clinical trials on IR from 1966 to 2014 were recorded compared with 27 clinical studies of single-implant placement [7], and evidence from clinical research of IR remains lacking [8]. In addition, it should be noted that the success/survival rate of IR was included in most of the previous studies; however, related factors such as tooth type and location, complications, and failures have seldom been reported [9]. Thus, the prognostic factors of IR should be addressed.

Recently, many researchers from several dental disciplines have been increasingly interested in IR with (bio)materials, including root-end biomaterials and periodontal regenerators [10,11]. Al-Hezaimi et al. [12] reported the successful treatment of a radicular groove by IR and Emdogain therapy. The results demonstrated that at 3-month recall, the patient was asymptomatic with a closed sinus tract. At 1-year follow-up, the patient was asymptomatic, exhibited active evidence of periodontal healing, comfortable pockets (≤3 mm), and a significant decrease in the size of the apical radiolucency. Demiralp et al. [3] reported that IR autologous platelet-rich plasma was used for the treatment of a periodontally involved, hopeless incisor. The patient achieved healthy gingiva and was satisfied with the outcome of the procedure. Moreover, no complications or postoperative pain or discomfort was noted.

Incorporating contemporary guidelines of tooth replantation and apical microsurgery into IR procedures may increase the potential for resorption-free reattachment and periapical healing. Recent clinical studies of IR report that long-term survival rates for patients can reach 73–77% [8,13]. This systematic review aimed to evaluate the clinical outcomes after IR of the teeth to determine the survival rate and prognostic factors related to the treatment.

## MATERIALS AND METHODS

### Search strategy

We searched several online databases (PubMed, OVID, and Cochrane databases) for relevant English language publications between January 2000 and October 2017. To maximize the identification of relevant articles, we used a population intervention comparison outcome (PICO) search strategy, including a combination of key words and/or MESH terms. The precise keywords used in our literature search included “intentional replantation” and “teeth”. An additional manual search was performed to identify relevant studies by screening the title and abstract of articles.

### Inclusion criteria and exclusion criteria

The publications were considered if all the following criteria were met: 1) English language publication between January 2000 and October 2017; 2) a minimum of 10 cases; 3) mean follow-up of at least 1 year; 4) reported details of IR; and 5) case series, randomized controlled trial (RCT), prospective or retrospective study type.

Studies not meeting all the inclusion criteria were excluded from the review. Publications dealing with the following topics were also excluded: animal studies, IR after traumatic injury, and compromised periodontal health/prognosis. In addition, we excluded case reports, expert opinions, review articles, or articles that exclusively focused on procedures.

### Literature selection and data extraction

Two reviewers independently screened the literature based on the inclusion criteria. First, the reviewers read the relevant abstracts from the literature. Second, to evaluate literature quality, the full texts were obtained, including the inclusion criteria, relevant information on the first author, publication year, type of study, number of cases, age and outcomes. Then, manual search was performed using the reference lists of the included studies to identify additional articles. Strict and uniform inclusion and exclusion standards were applied to select the literature, and two independent researchers used a blind method to reduce the selection bias. To thoroughly assess each included study and lower the within-study bias, the methodological index for non-randomized studies (MINORS) score standard [14] was applied for quality assessment. Each item in the MINORS has three scores: 0, unreported; 1, reported but inadequately or partially; and 2, adequately reported. All extracted data were double-checked, and any questions that arose during the screening and data extraction were discussed within the group to achieve a consensus. If consensus was not attained, a third reviewer served as an adjudicator.

### Definition of success and failure of operation

The outcomes can be defined as “success” or “failure”. IR was considered successful when patients exhibited no clinical symptoms, showed regeneration of the surrounding periodontal tissue radiographically and improvement in the periodontal probing depth at the fracture site. The following causes appearing in most studies were considered as failure: deeper pocket depth (>5 mm), pathologic tooth mobility, progressive root resorption, impertinent type of root-end filling material, and refracture.

## RESULTS

**FIGURE 1:**
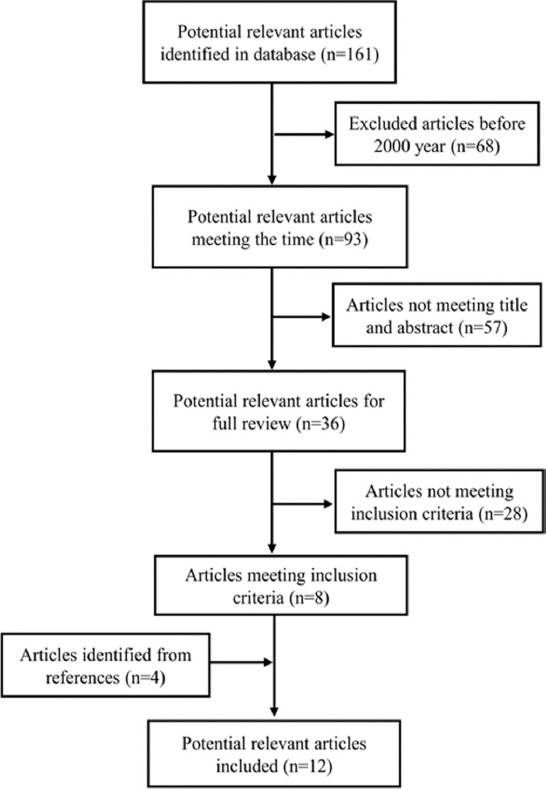
The flowchart of literature selection for the systematic review.

The electronic and manual search identified a total of 161 titles on intentional teeth replantation (for details, refer to Figure 1). In total, 68 and 57 studies were excluded due to irrelevant study period (before January 2000 or after October 2017) and irrelevant title and abstract, respectively. Then, 36 studies received full-text review. Among these studies, 28 studies were excluded because they were published in another language (n = 2), consisted of case reports (n = 14), contained insufficient data (n = 7), or involved animal teeth (n = 5). Furthermore, 4 studies from the references that met the inclusion criteria were included. Ultimately, 12 studies were included in the analysis (Figure 1). The search strategy was conducted using the PubMed, OVID, and Cochrane databases (Table 1). Table 2 presents the quality assessment of all eligible studies according to the MINORS score.

**TABLE 1:**
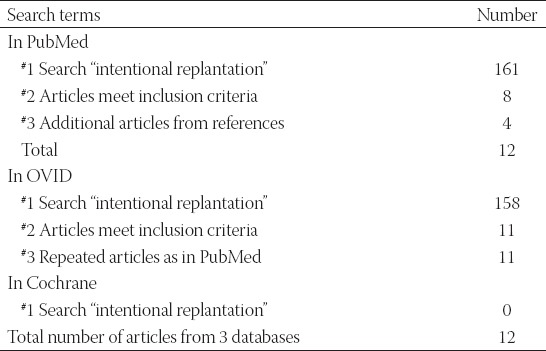
Search strategy in PubMed, OVID, and Cochrane databases

**TABLE 2:**
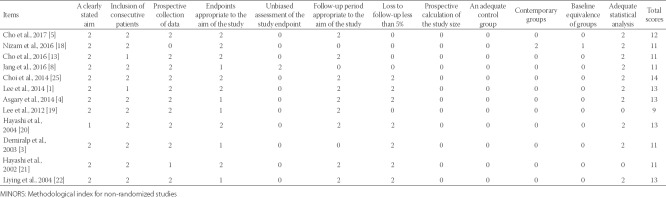
Quality assessment of all eligible studies according to the MINORS score

In the collected research papers, each study was a retrospective study and aimed to evaluate the clinical outcome of patients after IR. A total of 896 patients (905 teeth) from 12 studies underwent IR treatment. Three studies involved >100 teeth (25%), 3 studies involved ≤20 teeth (66.67%), and 6 studies involved between 20 and 70 teeth (41.67%). In the included studies, the patients’ ages ranged 11–75 years (mean 45 ± 10 years), and the follow-up time ranged 3–144 months. Eleven studies involved the teeth type and location (91.67%). The types of teeth included incisors, premolars, and molars. The locations were maxillary molar and mandibular molar, anterior and posterior teeth, second molars, and first molars. Nine studies (75%) of probing depth or pocket depths indicated that shallow probing depths were the most common. The extraoral times, as an important indicator of survival rate, were less than 15 minutes in 7 studies (58.33%). Nine studies involved root-end filling material (75%), mainly including intermediate restorative material (IRM)/Endocem/Super ethoxy benzoic acid (EBA)/ProRoot mineral trioxide aggregate [MTA] (4 studies, 44.44%) or 4-methacryloxyethyl trimellitate anhydride/methyl methacrylate-tri-n-butyl borane (META/MMA-TBB) dentin-bonded resin [3 studies, 33.33%] (Table 3).

**TABLE 3:**
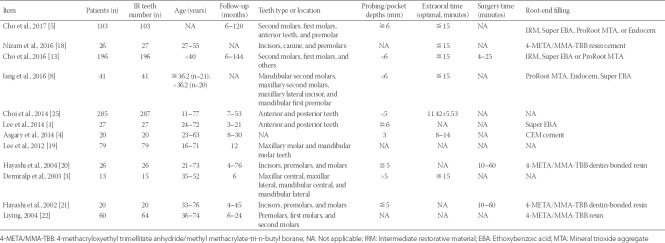
Study characteristics

In follow-up, the teeth rate referred to the tooth number within 6 months divided by the tooth number at IR operation. At 6-month follow-up, the subjects in 6 studies were not lost to follow-up. Three studies retained 80% of participants, and two retained 70%. The success rate in 4 studies was greater than 90%, and in 5 studies the rates were between 70% and 80%. In 6 studies, the reasons for failure were mainly attributed to refracture and abscess formation. During follow-up, complications including ankylosis, root resorption, abscess and pain were recorded in 7 studies. Among these studies, the patients in 3 studies presented with ankylosis symptoms, in 4 studies the patients experienced root resorption, and 3 studies reported abscess and pain after operation (Table 4).

**TABLE 4:**
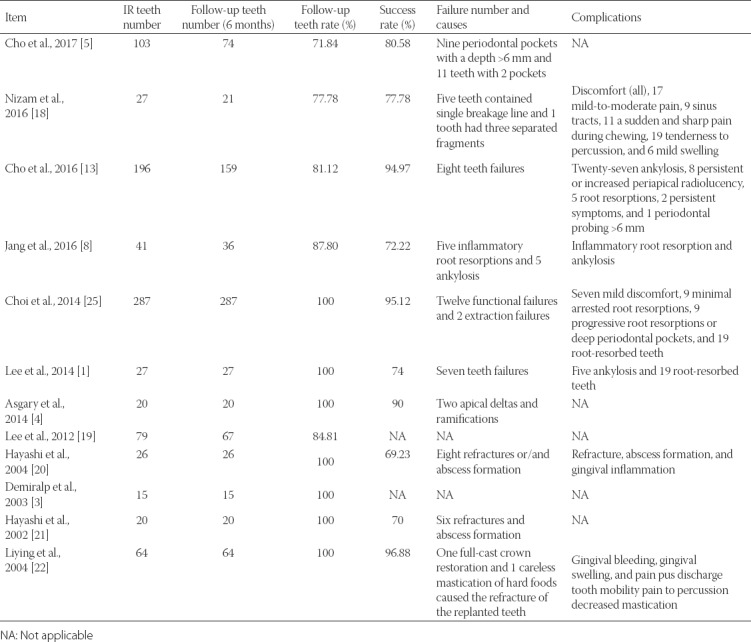
Success rate and complications

The survival rate was measured by Kaplan–Meier survival curves and expressed as a percentage (%). Ten studies (83.33%) reported survival rates over different follow-up periods. In 4 studies, the survival rate at short follow-up (<6 months) was quite high, at approximately 90%. At 6–12-month follow-up, the survival rate in 5 studies was 80–90%. At 12–36-month follow-up, the survival rates in 2 studies were reduced between 65% and 80%. One study reported that the survival rate of 83.3% at 12 months was reduced to 36.3% at 24 months. With longer follow-up (>36 months), the survival rates of teeth in 4 studies were slightly reduced and tended to be stable after 48 months. For the entire follow-up, we found that the survival rates were reduced as time progressed and stabilized at approximately 60% (Table 5).

**TABLE 5:**
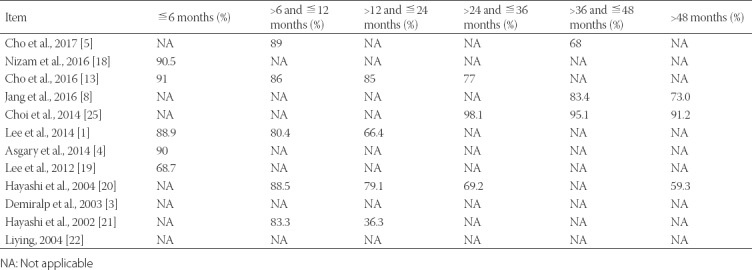
Survival rate

## DISCUSSION

This systematic review addressed the effect, survival rate and other prognostic factors of IR based on 12 included studies. The results of the meta-analysis demonstrated that long-term success and survival rate enhancement of IR are likely dependent upon short extraoral time, reductions in pocket depth, type of tooth, type of root-end material filling, and the prevention of atraumatic tooth root damage. It should be noted that although a small portion of patient data was lost in 5 studies, the follow-up data in the included studies were nearly complete. The follow-up rate was 100% in large samples and greater than 70% in small samples. The calculated success rate was greater than 70% in all studies and 90% in 4 studies. As presented in Table 5, the survival rate was as high as 90% in the short-term follow-up. Regarding the follow-up extension, the survival rate was reduced from 12 months to 36 months. When the follow-up was greater than 36 months, the survival rate was slightly reduced and tended to be stable. The long-term survival rate was not high, indicating that a high survival rate for teeth was difficult to maintain.

Periodontal and pulpal diseases are common and prevalent in natural teeth [15,16] and often simultaneously occur in the same tooth; they can be integrated and then form endodontic-periodontal lesions. Treatment of endodontic-periodontal lesions is a challenge because of the difficulty in diagnosis [5,15]. In endodontics, intentional teeth replantation is considered as the last treatment modality for apical periodontitis due to unfeasible non-surgical root canal treatment and periapical surgery [6,17]. The process of IR includes atraumatic tooth extraction, removal of local factors on both the tooth surface and extraction socket, and reinsertion of the tooth.

Many reported IR failures were attributed to a variety of reasons, mainly including complications and extraction operation, the type of fracture, pocket depth, and root-end filling materials. The complications after surgery occurred in different tooth parts such as the periodontal pocket, dental pulp and root, and included refracture, ramifications, root resorption and abscess formation, which are the main factors contributing to IR failures. For example, many studies reported vertical root fractures (VRFs) as a severe fracture type that might cause extensive injury of periodontal tissues, resulting in various complications including pain, swelling, sinus tract formation, increased tooth mobility, deep periodontal pockets and vertical bone resorption [4,8,18-22].

The extraction failures mainly resulted from difficult approach in periapical operation [13] and improper extraction time. A reduction in extraoral time is essential in the prevention of ankylosis and root resorption and the promotion of the periradicular healing process by preventing periodontal cell damage and dehydration [23]. Previous studies reported that extraoral time has been regarded as a significant factor affecting surgery results [8,13,18]. Cho et al. [13] considered that an extraoral time greater than 15 minutes increases the risk of complications and the occurrence of ankylosis. In addition, periradicular healing primarily depends on extraoral time. Pohl et al. [24] reported that if extraoral time is greater than 15 minutes for replanted teeth, root resorption might be expected to occur, and the risk of complications is 1.7-fold increased, thus reducing the survival rate of IR.

Some evidence indicates that tooth survival with healthy gingiva is associated with significant decreases in pocket depth [3]. Jang et al. [8] demonstrated that pocket depth was a representative indicator of periodontal condition and was determined by the maximum value of 6 measurements around the tooth. In the study of Choi et al. [25], normal physiologic mobility and moderate periodontal pocket depths (<5 mm) were confirmed with radiographs and periodontal probing. Renvert and Persson [26] performed a systematic review suggesting that the presence of residual probing depths >6 mm was associated with tooth disease progression.

The root-end filling material may also lead to IR failures. Hayashi et al. demonstrated that dentin-bonded resin cement that provides a sufficiently high bonding strength was a critical requirement for long-term success of reconstructed roots [20]. 4-META/MMA-TBB is a chemically cured resin cement that exhibits tolerance to the water content of dentine and surface moisture [27,28]. 4-META/MMA-TBB dentin-bonded resin has been used as the adhesive material given its superior adhesive property and biocompatibility. Nizam et al. [18] reported that the adhesive property was particularly important for reconstruction of fractured teeth because they were subject to continuous masticatory force. Among IRM/Endocem/Super EBA/ProRoot MTA filling materials, ProRoot MTA is more susceptible to early contamination and washout in IR teeth than Super EBA and Endocem [13].

Limitations in this systematic review were mainly due to incomplete data and a lack of studies on this research topic. First, inadequate indicators and incomplete data affected the assessment results and quality. Bone loss, periotest values (PTV), and gingival index (GI) were seldom mentioned in these studies, which severely reduced the quality of articles. The survival rate and surgery success rate were not referred to in some studies. Second, as noted in Table 5, the number of teeth cases is proportional to the survival rate. The more teeth, the higher the survival rate that can be achieved. However, many small cases were included in this systematic review, and the survival rate of small samples generally had poor accuracy and more errors. Long-term follow-up can improve survival rates. In addition, the selection of only English language studies in three databases was another limitation of this study. Finally, the quality of the included studies was poor mainly due to the missing groups for comparative analysis. Further studies on adequate indicators and additional cases are needed to improve the success of IR in clinical practice.

## CONCLUSION

The long-term success and survival rate enhancement of IR are likely dependent upon short extraoral time and reductions in pocket depth, type of tooth, type of root-end material filling, and the prevention of atraumatic tooth roots damage.

## References

[1] Lee EU, Lim HC, Lee JS, Jung UW, Kim US, Lee SJ (2014). Delayed intentional replantation of periodontally hopeless teeth:A retrospective study. J Periodontal Implant Sci.

[2] Lu DP (1986). Intentional replantation of periodontally involved and endodontically mistreated tooth. Oral Surg Oral Med Oral Pathol.

[3] Demiralp B, Nohutçu RM, Tepe DI, Eratalay K (2003). Intentional replantation for periodontally involved hopeless teeth. Dent Traumatol.

[4] Asgary S, Alim Marvasti L, Kolahdouzan A (2014). Indications and case series of intentional replantation of teeth. Iran Endod J.

[5] Cho SY, Lee SJ, Kim E (2017). Clinical outcomes after intentional replantation of periodontally involved teeth. J Endod.

[6] Grossman LI (1966). Intentional replantation of teeth. J Am Dent Assoc.

[7] Torabinejad M, Dinsbach NA, Turman M, Handysides R, Bahjri K, White SN (2015). Survival of intentionally replanted teeth and implant-supported single crowns:A Systematic review. J Endod.

[8] Jang Y, Lee SJ, Yoon TC, Roh BD, Kim E (2016). Survival rate of teeth with a C-shaped canal after intentional replantation:A Study of 41 cases for up to 11 years. J Endod.

[9] Raghoebar GM, Vissink A (1999). Results of intentional replantation of molars. J Oral Maxillofac Surg.

[10] Özer SY, Ünlü G, Değer Y (2011). Diagnosis and treatment of endodontically treated teeth with vertical root fracture:Three case reports with two-year follow-up. J Endod.

[11] Ozturk M, Unal GC (2008). A successful treatment of vertical root fracture:A case report and 4-year follow-up. Dent Traumatol.

[12] Al-Hezaimi K, Naghshbandi J, Simon JH, Rotstein I (2009). Successful treatment of a radicular groove by intentional replantation and emdogain therapy:Four years follow-up. Oral Surg Oral Med Oral Pathol Oral Radiol Endod.

[13] Cho SY, Lee Y, Shin SJ, Kim E, Jung IY, Friedman S (2016). Retention and healing outcomes after intentional replantation. J Endod.

[14] Slim K, Nini E, Forestier D, Kwiatkowski F, Panis Y, Chipponi J (2003). Methodological index for non-randomized studies (minors):Development and validation of a new instrument. ANZ J Surg.

[15] Figdor D (2002). Apical periodontitis:A very prevalent problem. Oral Surg Oral Med Oral Pathol Oral Radiol Endod.

[16] Chandra A, Yadav OP, Narula S, Dutta A (2016). Epidemiology of periodontal diseases in Indian population since last decade. J Int Soc Prev Community Dent.

[17] Bender IB, Rossman LE (1993). Intentional replantation of endodontically treated teeth. Oral Surg Oral Med Oral Pathol.

[18] Nizam N, Kaval ME, Gürlek Ö, Atila A, Çalışkan MK (2016). Intentional replantation of adhesively reattached vertically fractured maxillary single-rooted teeth. Int Endod J.

[19] Lee WC, Shon WJ, Baek SH, Kum KY, Kim HC (2012). Outcomes of intentionally replanted molars according to preoperative locations of periapical lesions and the teeth. J Dent Sci.

[20] Hayashi M, Kinomoto Y, Takeshige F, Ebisu S (2004). Prognosis of intentional replantation of vertically fractured roots reconstructed with dentin-bonded resin. J Endod.

[21] Hayashi M, Kinomoto Y, Miura M, Sato I, Takeshige F, Ebisu S (2002). Short-term evaluation of intentional replantation of vertically fractured roots reconstructed with dentin-bonded resin. J Endod.

[22] Liying Y, Beiyun X, Bin W, Qing C, Fang YR, Yamamoto K (2004). Clinical research on treatment of vertically fractured posterior teeth by intentional replantation using dentin bonding and composite resin. J Osaka Dent Univ.

[23] Nasjleti CE, Caffesse RG, Castelli WA (1978). Replantation of mature teeth without endodontics in monkeys. J Dent Res.

[24] Pohl Y, Filippi A, Kirschner H (2005). Results after replantation of avulsed permanent teeth II. Periodontal healing and the role of physiologic storage and antiresorptive-regenerative therapy. Dent Traumatol.

[25] Choi YH, Bae JH, Kim YK, Kim HY, Kim SK, Cho BH (2014). Clinical outcome of intentional replantation with preoperative orthodontic extrusion:A retrospective study. Int Endod J.

[26] Renvert S, Persson GR (2002). A systematic review on the use of residual probing depth, bleeding on probing and furcation status following initial periodontal therapy to predict further attachment and tooth loss. J Clin Periodontol.

[27] Tagami J, Tao L, Pashley DH (1990). Correlation among dentin depth, permeability, and bond strength of adhesive resins. Dent Mater.

[28] Tao L, Tagami J, Pashley DH (1991). Pulpal pressure and bond strengths of superBond and gluma. Am J Dent.

